# Upregulation of miR-99a is associated with poor prognosis of acute myeloid leukemia and promotes myeloid leukemia cell expansion

**DOI:** 10.18632/oncotarget.12947

**Published:** 2016-10-27

**Authors:** Xiaohui Si, Xiaoyun Zhang, Xing Hao, Yunan Li, Zizhen Chen, Yahui Ding, Hui Shi, Jie Bai, Yingdai Gao, Tao Cheng, Feng-Chun Yang, Yuan Zhou

**Affiliations:** ^1^ State Key Laboratory of Experimental Hematology, Institute of Hematology & Blood Diseases Hospital, Chinese Academy of Medical Sciences & Peking Union Medical College, Tianjin, China; ^2^ Center for Stem Cell Medicine, Chinese Academy of Medical Sciences, Tianjin, China; ^3^ Department of Stem Cell & Regenerative Medicine, Peking Union Medical College, Tianjin, China; ^4^ Collaborative Innovation Center for Cancer Medicine, Tianjin, China; ^5^ Sylvester Comprehensive Cancer Center, University of Miami Miller School of Medicine, Miami, FL, USA; ^6^ Department of Biochemistry and Molecular Biology, University of Miami Miller School of Medicine, Miami, FL, USA

**Keywords:** leukemia stem cell, microRNA, acute myeloid leukemia, miR-99a

## Abstract

Leukemia stem cells (LSCs) can resist available treatments that results in disease progression and/or relapse. To dissect the microRNA (miRNA) expression signature of relapse in acute myeloid leukemia (AML), miRNA array analysis was performed using enriched LSCs from paired bone marrow samples of an AML patient at different disease stages. We identified that miR-99a was significantly upregulated in the LSCs obtained at relapse compared to the LSCs collected at the time of initial diagnosis. We also found that miR-99a was upregulated in LSCs compared to non-LSCs in a larger cohort of AML patients, and higher expression levels of miR-99a were significantly correlated with worse overall survival and event-free survival in these AML patients. Ectopic expression of miR-99a led to increased colony forming ability and expansion in myeloid leukemia cells after exposure to chemotherapeutic drugs *in vitro* and *in vivo*, partially due to overcoming of chemotherapeutic agent-mediated cell cycle arrest. Gene profiling and bioinformatic analyses indicated that ectopic expression of miR-99a significantly upregulated genes that are critical for LSC maintenance, cell cycle, and downstream targets of E2F and MYC. This study suggests that miR-99a has a novel role and potential use as a biomarker in myeloid leukemia progression.

## INTRODUCTION

Acute myeloid leukemia (AML) is a genetically and clinically heterogeneous disease driven by a subpopulation of leukemia stem cells (LSCs) with self-renewal properties that generate the bulk of leukemia cells [1–3]. LSCs originate from hematopoietic stem cells (HSCs) or more lineage-restricted progenitors, which have become leukemic due to accumulated mutations [4, 5]. Like their HSC counterparts, LSCs are at the top of the hierarchical organization of AML [6]. However, LSCs can resist available treatments and thus act as barriers to cure, leading to the progression and relapse of AML following chemotherapy [7]. The high number of LSCs or expression of an LSC related gene signature is independently associated with poor prognosis in AML, supporting the notion for LSCs as important targets for therapeutic intervention [1, 8, 9].

Epigenetic modifications such as DNA methylation, histone modifications and microRNAs (miRNAs), contribute significantly to the initiation, progression and/or prognosis of leukemia [8]. MiRNAs are a family of 21-25-nucleotide small RNAs that negatively regulate gene expression at the post-transcriptional level [10]. These small non-protein-coding RNAs are critical regulators of many physiological processes such as cell proliferation, apoptosis and differentiation [11]. Emerging evidence suggests that dysregulated expression of miRNAs significantly involved in various cancers [12, 13] including leukemia [14, 15]. As an example, miR-17-92 polycistron is particularly overexpressed in AML bearing mixed lineage leukemia (MLL) gene rearrangements and maintains LSC self-renewal [16, 17]. Overexpression of miR-155 initiates expansion of preleukemic pre-B cells, leading to the development of frank B cell malignancy in a transgenic mouse model [18]. Thus, miRNAs have begun to garner attention as potential targets for the treatment of leukemia.

In this study, relapse-related miRNA expression signature was firstly analyzed by miRNA array in enriched CD34^+^ LSCs obtained from paired bone marrow (BM) samples from an AML patient at initial diagnosis and relapse, and miR-99a was the most significantly upregulated miRNA in LSCs at relapse stage compared to the initial diagnosis. We also observed that miR-99a was highly amplified in LSCs compared to non-LSCs in a cohort of 18 AML patients, and the high expression levels of miR-99a were associated with the poor prognosis of patients with AML. These results point to the importance of a novel role of miR-99a in LSC activity and disease prognosis.

## RESULTS

### Upregulation of miR-99a in LSCs was associated with poor prognosis of AML

**Figure 1:**
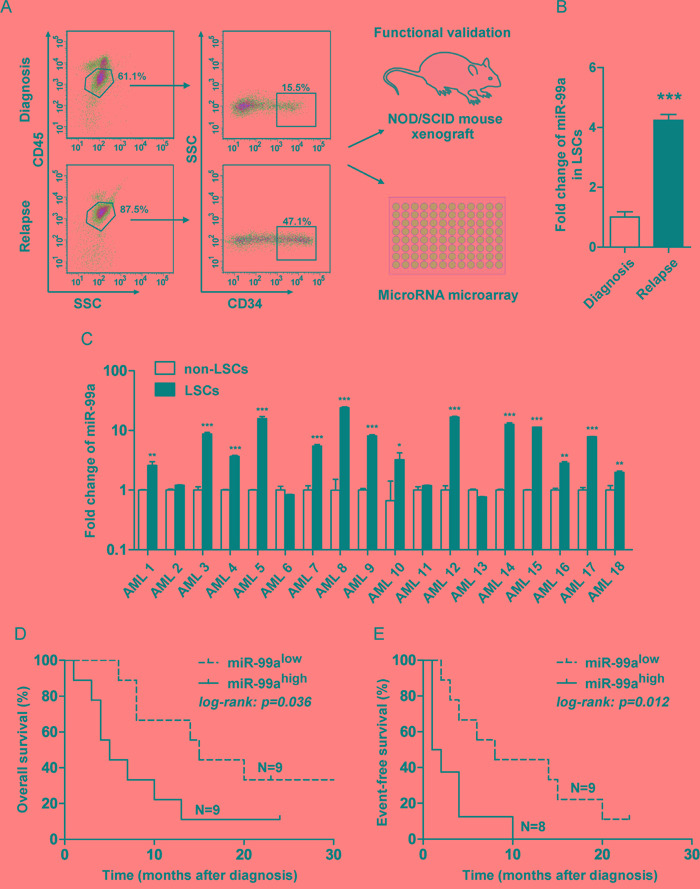
Upregulation of miR-99a in LSCs was associated with poor prognosis of AML **A.** Strategy to fractionate AML patient samples based on SSC and immuno-phenotypic staining with CD34 and CD45. LSCs were enriched as SSC^low^CD45^dim^CD34^+^, and non-LSCs were enriched as SSC^low^CD45^dim^CD34^−^. Functional validation of LSC-containing fractions was performed by xenotransplantation. MiRNA array of LSCs was analyzed to generate relapse related miRNA expression profile in LSCs. **B.** Fold changes of miR-99a in paired LSCs obtained at relapse or initial diagnosis were validated by qPCR. Data are presented as mean ± SD, and represented triplicate wells from one of three independent experiments. U6 was used as the endogenous reference gene. ***p<0.001. **C.** Differential expression of miR-99a between LSCs and non-LSCs in 18 AML patients by qPCR analyses. The results were expressed as the fold change of LSCs relative to non-LSCs. Data are presented as mean ± SD, and represented triplicate wells from one of two independent experiments. U6 was used as the endogenous reference gene. *p<0.05, **p<0.01, ***p<0.001. **D-E.** Correlation between miR-99a expression level with OS (D) and EFS (E) in patients with AML. The subjects were divided into two group basis on the median expression of miR-99a in LSCs reference to non-LSCs. OS and EFS were estimated using the Kaplan-Meier analyses and a log-rank test.

LSCs were initially thought to reside in the CD34^+^CD38^−^ cell population [19, 20]. However, with improved xenotransplantation model, LSCs were also detected in the CD34^+^CD38^+^ cell fraction of some subjects previously thought to be non-LSCs [1, 7]. In this study, LSCs and non-LSCs were distinguished from leukemic blast cells by flow cytometry based on the surface expression of CD34. LSCs from paired BM of an AML patient at diagnosis and relapse were enriched as SSC^low^CD45^dim^CD34^+^ (Figure 1A), and then validated for the characteristic feature of the LSCs by an *in vivo* xenotransplantation assay (Figure S1A-S1B). The paired LSCs were subsequently used for miRNA array analysis. MiRNA array analysis revealed that a series of miRNAs were upregulated in the LSCs obtained at relapse compared to the LSCs collected at the time of initial diagnosis, and quantitative real-time PCR (qPCR) assays revealed that miR-99a was the most significantly differential miRNAs among the upregulated miRNAs in LSCs at relapse (Figure S1C and 1B). Since LSCs are supposedly responsible for the outcome of both leukemia initiation and relapse, we performed qPCR analyses to validate the differential expression of miR-99a in paired LSC and non-LSC subpopulations from a cohort of 18 AML patients at initial diagnosis. The results revealed that miR-99a was significantly overexpressed in LSCs compared to paired non-LSCs in 14 out of 18 AML patients (Figure 1C). The median increase of miR-99a expression was 3.7-folds in LSC/non-LSCs, while that was only 1.2-folds in CD34^+^ cells compared to CD34^−^ cells sorted from cord blood (CB) of healthy donors (Figure S1D). Moreover, the expression level of miR-99a was markedly higher in KG-1a and KG-1 cells than in other myeloid leukemia cell lines (Figure S1E). Of note, both KG-1a and KG-1 cells express human hematopoietic stem and progenitor cell antigen CD34, and are considered as the most primitive myeloid leukemia cell lines [21–23]. To determine whether miR-99a overexpression in LSCs correlated with the prognosis of AML, we divided the subjects into two groups based on the median expression level of miR-99a (miR-99a^high^ and miR-99a^low^). Kaplan-Meier analysis and the log-rank test revealed that upregulated miR-99a significantly correlated with worse overall survival (OS) (Figure 1D) and event-free survival (EFS) (Figure 1E). The median of OS was 4 months in miR-99a^high^ group compared to 13 months in miR-99a^low^ group, and additionally, the median of EFS was 1 month in miR-99a^high^ group compared to 9 months in miR-99a^low^ group, which is consistent with the finding that miR-99a is upregulated in LSCs at relapse stage compared to the paired new-diagnostic stage by miRNA array.

To determine if the increased level of miR-99a in LSCs correlated with resistance to chemotherapy, we compared the miRNA levels in the resistant derivatives of K562 cells (a multidrug-resistant derivative of K562 cells, K562/A02, and an imatinib-resistant derivative of K562 cells, K562/G01), to the parental cells. Our results revealed that miR-99a were significantly upregulated in K562/A02 cells and K562/G01 cells than K562 cells (Figure S1F). These data suggest that higher level of miR-99a may associate with the resistance of chemotherapy.

### Ectopic miR-99a expression resulted in increased colony forming ability in primary AML LSCs

**Figure 2:**
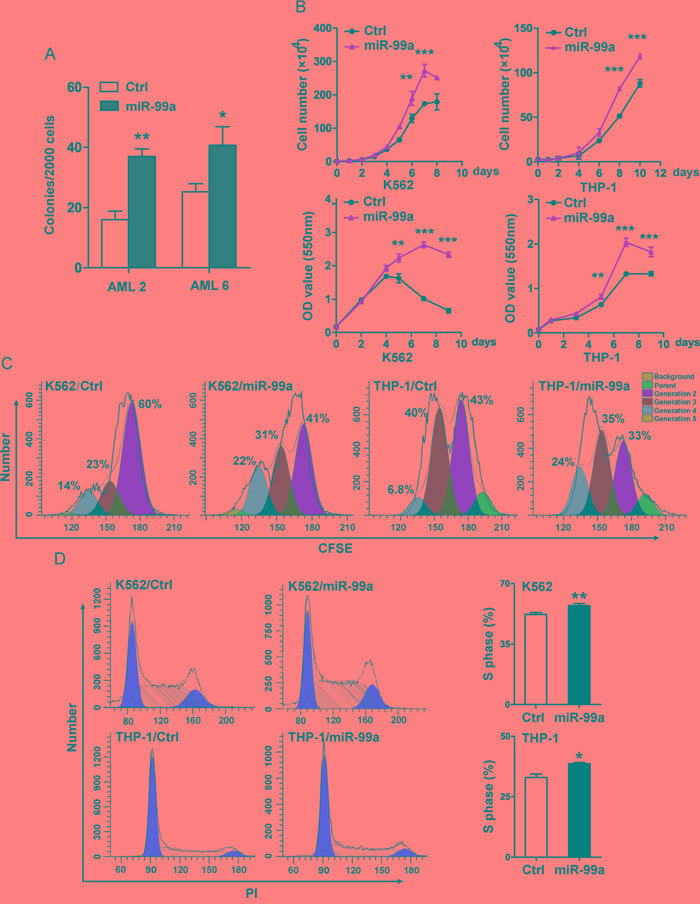
Ectopic miR-99a expression accelerated the growth of myeloid leukemia cells **A.** Colony forming ability of primary AML samples with ectopic miR-99a expression. LSCs enriched from AML patients (AML2 and AML6) were transduced with miR-99a or Ctrl viruses, eGFP positive cells were sorted to evaluate the colony forming ability. Colony numbers were scored at day 14. Data are presented as mean ± SD, and represented one of two independent experiments. *p<0.05, **p<0.01. **B.** Proliferation/Viability of K562 and THP-1 cells transduced with Ctrl or miR-99a viruses were measured by manually counting (upper panel) and MTT staining (lower panel). Data are presented as mean ± SD from three individual experiments. **p<0.01, ***p<0.001. **C.** Cell division of K562 and THP-1 cells transduced with Ctrl or miR-99a viruses were measured by CFSE staining. Cells were collected at different time points (24 hrs for K562 cells, 48 hrs for THP-1 cells) after CFSE dye labeling, and estimated by flow cytometry. Cells were collected at 0 hr post dye staining as an undivided control. The discrete peaks in this histogram represented successive generations of live cells. Data represented one of two independent experiments. **D.** Representative flow cytometry histograms show the cell-cycle distribution of K562 and THP-1 cells transduced with Ctrl or miR-99a viruses which were measured by PI staining (left panel). Quantitation of cell-cycle distribution of S-phase is shown (right panel). Data are presented as mean ± SD from three individual experiments. *p<0.05, **p<0.01.

To determine the potential effects of miR-99a upregulation on cellular function of LSCs, primary CD34^+^ cells were isolated from two AML patients with a low expression level of miR-99a (AML2 and AML6, as shown in Figure 1C) then transduced with lentivirus carrying hsa-miR-99a-5p (miR-99a) or a scrambled sequence (Ctrl), tagged with enhanced green fluorescent protein (eGFP) (Figure S2A), followed by the colony forming cell (CFC) assay. The result showed a 1.6- and 2.3-fold increase in the number of colonies after ectopic expression of miR-99a in the two AML patients, respectively (Figure 2A), indicating that upregulation of miR-99a enhanced the colony forming activity of LSCs.

### Ectopic miR-99a expression accelerated the growth of myeloid leukemia cells

We next transduced K562 and THP-1 cells with miR-99a or Ctrl vectors and measured the levels of miR-99a by qPCR (Figure S2B). Cell proliferation was measured by manually cell counting, and was further confirmed by MTT assay. Ectopic expression of miR-99a significantly accelerated the growth of both K562 and THP-1 cells (Figure 2B). The estimated cell doubling time for miR-99a and Ctrl cells was 26.6 ± 0.2 hrs and 32.35 ± 0.4 hrs in K562 cells, and 47.3 ± 0.2 hrs and 54.6 ± 0.07 hrs in THP-1 cells, respectively. CFSE analyses were conducted to monitor distinct generations of proliferating cells by CFSE dye dilution. Twenty-four hours after CFSE labeling, the proportions of K562/miR-99a cells in the third and fourth generation were almost doubled compared to K562/Ctrl cells (Figure 2C). A similar accelerated cell division was also observed in THP-1/miR-99a cells compared to THP-1/Ctrl cells after 48 hrs (Figure 2C). Additionally, cell cycle analyses showed that ectopic expression of miR-99a increased the proportion of cells in the S-phase in both K562 and THP-1 cells (Figure 2D). These data suggest that ectopic expression of miR-99a accelerated the growth of myeloid leukemia cells *in vitro*.

### Ectopic miR-99a expression promotes leukemic cell survival after exposure to chemotherapeutic agents *in vitro*

**Figure 3:**
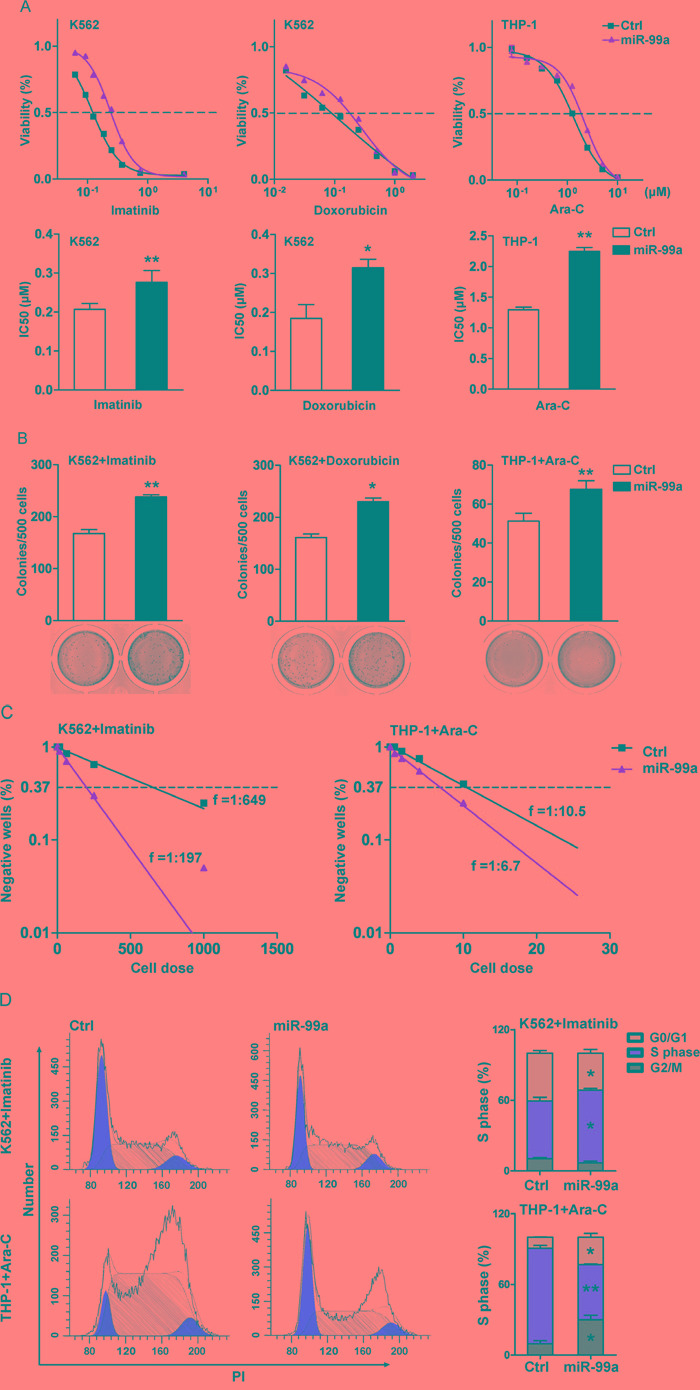
Ectopic miR-99a expression promotes leukemic cell survival after exposure to chemotherapeutic agents *in vitro* **A.** K562 and THP-1 cells transduced with miR-99a and Ctrl viruses were treated with increasing concentrations of imatinib, doxorubicin, or Ara-C for 72 hrs, respectively. Results were expressed as viability percentage relative to the untreated group. IC_50_ values were calculated with GraphPad Prism software, which was marked in the middle of the bottom and top plateau. Data are presented as mean ± SD, and represented one of four independent experiments. *p<0.05, **p<0.01. **B.** CFC assays of cells after drug treatment. K562/miR-99a and K562/Ctrl cells were exposed to 0.12 μM imatinib or 0.02 μM doxorubicin for 24 hrs, THP-1/miR-99a and THP-1/Ctrl cells were exposed to 1.25 μM AraC for 24 hrs, and followed by culturing in methylcellulose medium (7 days for K562 and ten days for THP-1). Data are presented as mean ± SD, and represented one of three independent experiments. *p<0.05, **p<0.01. **C.** The frequency of resistant cells was estimated by limiting-dilution assay. K562/miR-99a and K562/Ctrl cells were exposed to 0.25 μM imatinib for 14 consecutive days. THP-1/miR-99a and THP-1/Ctrl cells were previously incubated for 24 hrs with 1.25 μM Ara-C, washed, and then cultured for another 14 days. Individual wells were considered negative if there was no cell growth after two weeks. Data represented one of two independent experiments. **D.** Cell-cycle distribution of cells after drug treatment. K562 and THP-1 cells transduced with miR-99a and Ctrl viruses were treated with imatinib and Ara-C, respectively. Representative flow cytometry histograms show the cell-cycle distribution in cells posts treatment by PI staining (left panel). Quantitation of cell-cycle distribution is shown in (right panel). Data are presented as mean ± SD, and represented one of three individual experiments. *p<0.05, **p<0.01.

To further determine if the elevated level of miR-99a is a cause or a result of chemoresistance, we next examined the sensitivity to various chemotherapeutic agents in miR-99a overexpressed K562 and THP-1 cells. Transduction of miR-99a into K562 cells led to a 2.3~ and 2~folds increase in the IC_50_ to imatinib and doxorubicin compared to K562/Ctrl cells (Figure 3A). Similarly, ectopic expression of miR-99a markedly increased the survival rate of THP-1 cells in the culture containing cytarabine (Ara-C) for 72 hrs (Figure 3A). Consistently, CFC assay showed that ectopic expression of miR-99a increased the colony forming ability of K562 or THP-1 cells that were exposed to imatinib, doxorubicin, or Ara-C for 24 hrs (Figure 3B).

To assess the frequency of resistant clones following exposure to chemotherapeutic agents, we next performed limiting dilution analyses on the K562 or THP-1 cells transduced with either miR-99a or Ctrl vector. The frequency of resistant cells was 1:197 and 1:649 in K562/miR-99a and K562/Ctrl cells in the presence of 0.25 μM imatinib consecutively for 14 days. The frequency was 1:6.7 and 1:10.5 in THP-1/miR-99a and THP-1/Ctrl cells after exposure to 1.25 μM Ara-C for only 24 hrs followed by a 14-days of culture, respectively (Figure 3C). Ara-C is an antimetabolite antineoplastic agent that inhibits the synthesis of DNA [24, 25]. Ara-C treatment induced significant S-phase arrest in THP-1/Ctrl cells, while THP-1/miR-99a cells showed less S-phase arrest 24 hrs post treatment compared to untreated cells (Figure 2D, 3D). Different from Ara-C, imatinib can induce cell cycle arrest at G1 phase [26], and K562/miR-99a cells still showed less G1 arrest compared to K562/Ctrl cells after treated with imatinib for 24 hrs (Figure 2D, 3D). However, the results of apoptosis analyses showed no significant difference between miR-99a and Ctrl cells after exposure to these chemotherapeutic agents (Figure S3). Collectively, ectopic expression of miR-99a in myeloid leukemia cells resulted in increased cell survival after chemotherapy *in vitro* as reflected by the increased colony forming ability and the higher frequency of resistant cells, which are likely due to overcoming cell cycle arrest induced by chemotherapeutic drugs.

### Ectopic miR-99a expression promotes leukemic cell survival after exposure to chemotherapeutic agents *in vivo*

**Figure 4:**
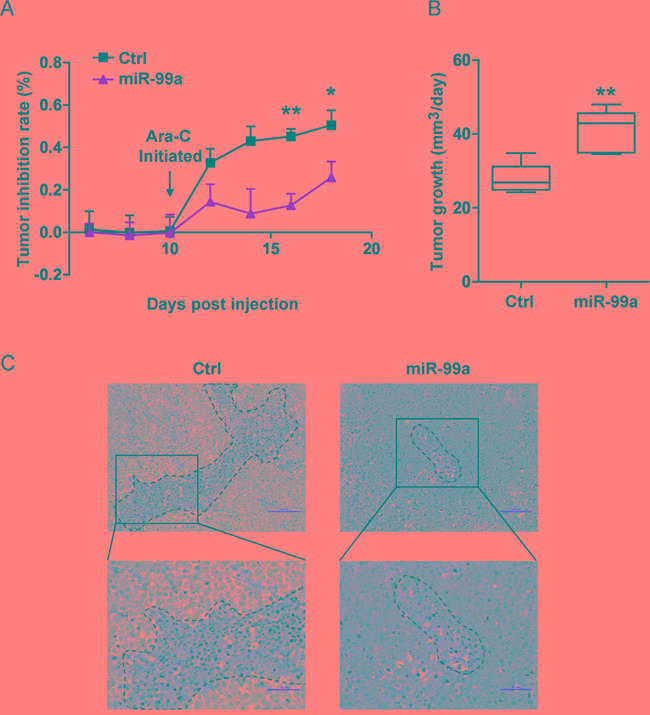
Ectopic miR-99a expression promotes leukemic cell survival after exposure to chemotherapeutic agents *in vivo* **A-B.** Nude mice bearing THP-1/Ctrl and THP-1/miR-99a cells were treated with Ara-C daily at 50 mg/kg for a week and tumor inhibition rate was measured based on tumor volumes of Ara-C and control group every other day (A). The tumor growth of THP-1/Ctrl and THP-1/miR-99a cells post Ara-C treatment was shown in (B), which was defined as the increase in tumor volume per day (mm^3^/day). Data are presented as mean ± SD (n=5 mice/group), and represented one of two independent experiments. *p<0.05, **p<0.01. **C.** Histological sections of tumors stained with H&E. Representative images of the tumor from Ctrl or miR-99a mice were taken under 20× magnification (upper panel) and 40× magnification (lower panel). Scale bars represent 100 μm (upper panel) and 50 μm (upper panel). Necrotic areas are indicated by dotted lines.

To determine the potential role of miR-99a in chemoresistance *in vivo*, we injected THP-1/miR-99a and THP-1/Ctrl cells subcutaneously into nude mice. When tumor reached 200 mm^3^, mice were treated with Ara-C daily at 50 mg/kg for a consecutive 7 days. The tumor volume was measured every other day, and the tumor inhibition rate was calculated based on tumor volume of Ara-C treatment group relative to control group. A significant lower tumor inhibition rate was observed overtime in the THP-1/miR-99a cell injected mice compared to the THP-1/Ctrl cell injected mice (Figure 4A). On the other hand, a mean of 40.8 mm^3^/day of tumor growth was observed in the mice received THP-1/miR-99a cells, while a mean of 27.8 mm^3^/day of tumor growth was observed in the mice injected with THP-1/Ctrl cells during the treatment (Figure 4B). H&E staining of xenograft tumors from nude mice revealed that the Ctrl group had more necrotic cells than miR-99a group after Ara-C treatment (Figure 4C). Taken together, ectopic expression of miR-99a promotes leukemic cell survival after exposure to chemotherapeutic agents *in vivo*.

### Gene profiling analyses showed dysregulation of leukemic expansion associated gene upon miR-99a overexpression

**Figure 5:**
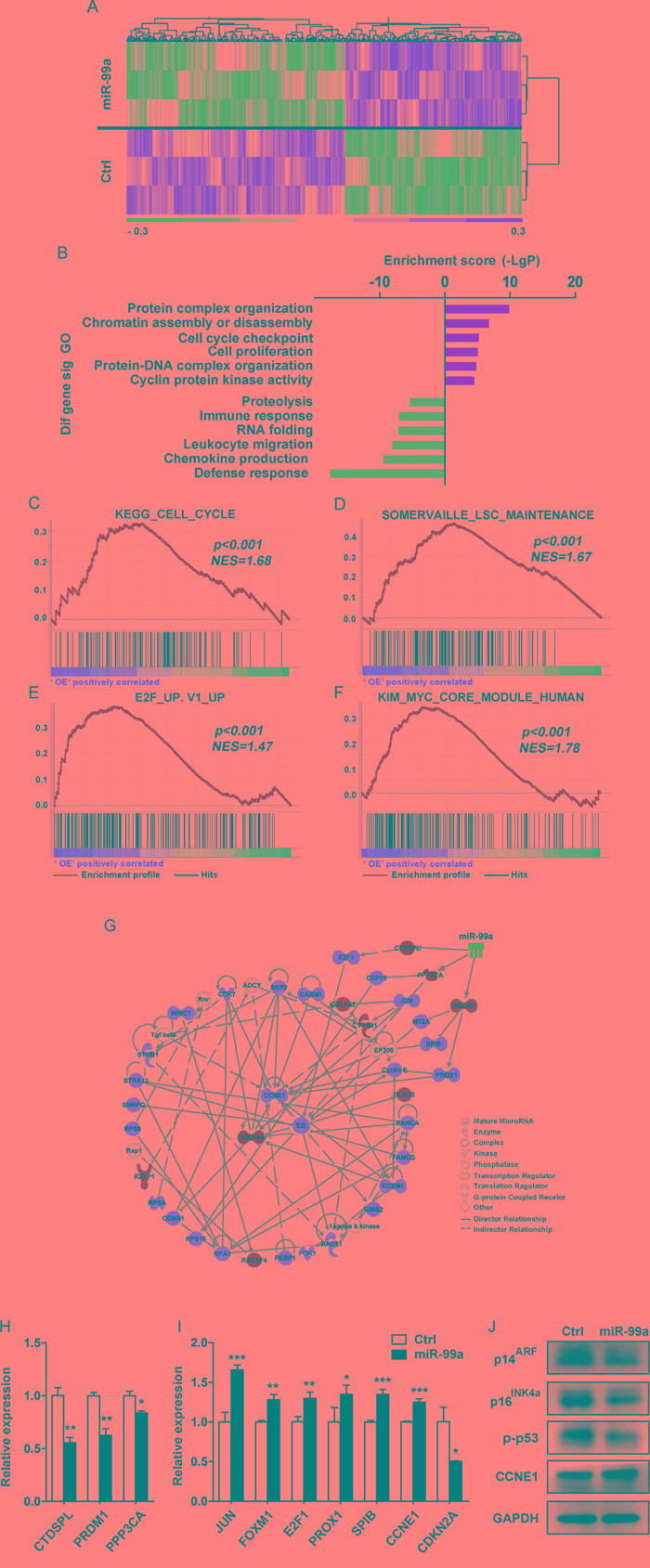
Gene profiling analyses showed differentially expressed gene upon miR-99a overexpression **A.** Heat map of microarray analysis of the significantly different expression levels of mRNA between THP-1/Ctrl and THP-1/miR-99a groups. (n=3 per group, *p*<0.05, fold-change ≥1.2). **B.** GO analysis of microarray data. Selected significant ontology terms are shown. **C-F.** GSEA analyses of gene expression profile in miR-99a versus Ctrl cells. The enrichment score plots corresponded to the KEGG cell cycle gene set (C), the LSC maintenance signature gene set (D), E2F-induced upregulated gene set (E), and the MYC core module gene set (F). The normalized enrichment score (NES) and *p*-values are shown. **G.** Representative protein-protein interaction network of proliferation/survival related genes based on the IPA database. Node shape corresponds to molecular function type. Node color corresponds to up-regulation (red) or down-regulation (green). Visualization was done using IPA software. **H-J.** Relative expression levels of miR-99a candidate targets (H), key downstream genes (I) were confirmed by qPCR and protein expression of relevant differentially expressed genes was analyzed by Western blot (J). Data are presented as mean ± SD and represented one of two independent experiments. *p<0.05, **p<0.01, ***p<0.001.

To identify gene candidates that may account for the observed cellular phenotype such as accelerated cell growth and increased drug resistance mediated by miR-99a, microarray assay was performed in THP-1 cells transduced with miR-99a or Ctrl viruses, respectively. A total of 776 differentially expressed genes (356 upregulated and 420 downregulated) were identified in THP-1/miR-99a compared to THP-1/Ctrl cells (Figure 5A). Gene Ontology (GO) analyses revealed that the differential genes were mainly enriched in 49 up-regulated GO terms and 71 down-regulated GO terms in THP-1/miR-99a cells. The upregulated GO terms mediated by miR-99a mainly included categories of protein complex organization, chromatin remodeling, cell cycle regulation and cell proliferation, while categories of proteolysis, immune response and RNA folding were significantly downregulated (Figure 5B). Pathway enrichment analyses showed that the differential genes were mainly involved in 14 significantly up-regulated pathways and 15 significantly down-regulated pathways (Figure S4A-S4B). Moreover, Gene set enrichment analysis (GSEA) revealed significant enrichment of KEGG cell cycle genes, E2F target genes, MYC target genes, and LSC maintenance associated genes in cells with ectopic miR-99a expression (Figure 5C-5F). Heat maps of the enriched genes were shown in Figure S4C-S4F. On the other hand, genes related to inflammatory response and interferon alpha response were enriched in Ctrl group (Figure S4G-S4H). Differential gene interactions in the Signal-Net were analyzed by the Ingenuity Pathway Analysis (IPA), and *E2F*, *CCNE1* and *CDKN2A* were observed as the core genes of the regulation network downstream of miR-99a (Figure 5G). Several predicted targets of miR-99a were found to be significantly downregulated in microarray analysis, including *BMPR2, CTDSPL, GPR26, KBTBD8, NIPBL, PPP3CA, PRDM1, TMPRSS13*, and *SMARCA5*. Among these, *CTDSPL, PRDM1* and *PPP3CA* were the candidate targets of miR-99a as they closely linked to proliferation and survival network based on IPA network analysis (Figure 5G). The expression of the candidate targets and key downstream genes of miR-99a was further confirmed by qPCR (Figure 5H-5I). Western blot analyses further demonstrated the upregulation of CCNE1 and the downregulation of p14^ARF^, p16^INK4a^ (two proteins encoded by CDKN2A) and phospho-p53 (p-p53) (Figure 5J).

## DISCUSSION

LSCs are hypothesized to underlie leukemic initiation, progression and relapse as they can reacquire the stem cell capability of self-renewal and generate non-LSCs [19, 20]. The high expression of LSC related signature genes was negatively correlated with the complete remission [1]. MiRNAs have been shown to be involved in various physiological and pathological processes, such as cell proliferation, differentiation, metabolism and cancer progression [11, 27]. There are several studies investigating the miRNA profiles in primary AML cells [28–30], but few studies have focused on LSCs. It is important to explore the potential role of LSC-related miRNAs in maintaining LSC properties, such as self-renewal and therapy resistance, which are vital for the progression and prognosis of AML. In this study, we compared for the first time the miRNA expression profiles in LSCs obtained from paired BM samples of an AML patient at diagnosis and relapse. Relapse-associated miRNA signature has been identified, and miR-99a was found to be the most significant differentially expressed miRNA. By comparing miRNA expression profiles of the same patient at different disease stages, the effect of individual differences can be reduced, and thus only disease progression related miRNAs are enriched and identified. We further examined miR-99a expression in LSCs and non-LSCs obtained from 18 AML patients at initial diagnosis, and found that miR-99a was significantly overexpressed in LSCs compared to non-LSCs in 14/18 patients. Importantly, we found that OS and EFS were significantly worse in patients with higher miR-99a levels, thus unveiling that high miR-99a expression correlates with poor prognosis including relapse and therapy resistance in AML patients. However, further studies with a larger cohort are warranted to validate our findings. Similarly, Zhang et al. showed that miR-99a expression decreased sharply in 91% whole BM samples from patients with pediatric AML and Chronic myeloid leukemia (CML) at complete remission compared to that at disease diagnosis [31]. However, miR-99a has been reported to works as a tumor suppressor and downregulated in a variety of human cancers, such as breast cancer [32], cervical cancer [33], prostate cancer [34] and osteosarcoma [35]. The finding that miR-99a plays opposing roles in human myeloid leukemia versus solid tumor suggesting the tissue-specific features as well as complexities of the regulation network of miRNAs, which is consistent with the previous studies [36–39].

Our studies demonstrated that ectopic expression of miR-99a increased the clonogenic capacity of primary AML LSCs. Further studies in myeloid leukemia cell lines revealed that ectopic miR-99a led to accelerated cell-cycle progression and cell proliferation. MiR-99a combined with miR-125b has been reported to induce resistance to vincristine in ETV6-RUNX1-positive acute lymphocytic leukemia [40]. However, the role of miR-99a in drug resistance in myeloid leukaemia has not been well documented. In our study, miR-99a overexpression in K562 and THP1 cells led to decreased sensitivity to multiple first-line chemotherapeutic agents such as imatinib and Ara-C *in vitro*, promoted cell survival after exposure to drugs. More importantly, miR-99a overexpression resulted in a resistant to Ara-C by a xenograft model *in vivo*. The miR-99a mediated drug resistance is likely, at least in part, due to the overcoming of cell cycle arrest. In addition, higher miR-99a expression levels were found in doxorubicin and imatinib resistant K562 cell lines (K562/A02 and K562/G01) compared to their parental cell lines. All these results demonstrated that miR-99a, the miRNA enriched from LSCs at relapse stage, provides a survival advantage and promotes the expansion of myeloid leukemia cell.

To explore the mechanism underlying the enhanced proliferation and chemoresistance meditated by miR-99a, we identified differential gene expression profiles by microarray analysis. The results provide a potential regulatory pathway that links the downregulated targets of miR-99a (i.e., *CTDSPL, PRDM1* and *PPP3CA*) to the dysregulated key genes associated with leukemic cell expansion. For example, miR-99a overexpression in THP-1 cells upregulated genes encoding key transcriptional factors in hematopoietic malignancies such as *JUN*, and cell cycle regulators such as *E2F1* and *FOXM1*. Additionally, overexpression of miR-99a led to downregulation of the *CDKN2A*, which encodes p16^INK4a^ and p14^ARF^ and mediate G1 cell cycle arrest by stabilizing the structure of p53 or inducing p53 activation. All these results suggest an important role of miR-99a in cell cycle entry and progression, leading to the accelerated proliferation and overcoming of chemotherapeutic-agents mediated cell cycle arrest.

Pathway and GO terms revealed that the genes associated with the metabolic process were enriched in miR-99a overexpressed cells, such as *ATP1B1*, *ATP5G1* and *ATP5EP2*. Katajisto et al. reported that newly produced mitochondria can maintain stem properties [41]. Our data that overexpression of miR-99a increases the expression of the genes critical for cell cycle, DNA repair, metabolic process and protein complex organization, indicates that miR-99a may promote cell division by enabling cells to acquire new organelles and proteins. It is possible that higher miR-99a levels in leukemia cells may lead to the acquisition of LSC-like traits. Here, we show that overexpression of miR-99a upregulates MYC target genes, cell cycle regulators, and LSC maintenance associated genes. Dysregulation of these miR-99a-associated genes results in a cellular characteristic with both competitive advantage and LSC maintenance. In fact, this is consistent with the finding by Laurenti et al. that accelerating exit from quiescence did not alter the balance between self-renewal and differentiation or impair stem cell maintenance [42].

Collectively, our study provides strong evidence that higher levels of miR-99a are associated with poor prognosis of AML that results in cell expansion and progression of myeloid leukemia. In addition, the expression level of miR-99a may be used as a biomarker for the prognosis and progression of AML.

## MATERIALS AND METHODS

### Human subjects and cell lines

BM samples and medical histories were obtained from a cohort of 18 individuals with AML (aged 18-70 years) at Institute of Hematology and Blood Diseases Hospital, Chinese Academy of Medical Sciences from 2007 to 2014. All patients signed the informed consent form, and the study protocol was approved by the Ethics Committee of our hospital, conforming to the ethical guidelines of the 1975 Helsinki Declaration. All patients were reevaluated and met 2008 WHO diagnostic criteria for AML. Mononuclear cells were isolated by standard ficoll procedure and frozen viably.

BM mononuclear cells (MNCs) were stained with fluorochrome-conjugated monoclonal antibodies, and sorted by flow cytometry. LSCs were enriched as side scatter (SSC)^low^CD45^dim^CD34^+^, and non-LSC leukemia blast cells as SSC^low^CD45^dim^CD34^−^, respectively. DAPI (Sigma-Aldrich, St Louis, USA) was used to exclude dead cells. Antibodies were purchased from BD Biosciences, including anti-CD45 (FITC), anti-CD34 (PE), and anti-CD33 (PE-Cy7). CB hematopoietic stem and progenitor cells obtained from consenting healthy donors were enriched using CD34^+^ magnetic beads (Miltenyi, Bergisch Gladbach, Germany).

Human hematopoietic malignant cell lines used in this study were purchased from American Type Culture Collection. K562, U937, NB4, and THP-1 cells were cultured in RPMI 1640 (Gibco, Carlsbad, USA) supplemented with 10% fetal bovine serum (FBS, Gibco) and 1% penicillin/streptomycin (Beyotime, Shanghai, China). K562/A02 induced by doxorubicin were cultured in the same complete RPMI 1640 media containing 1.5 μM doxorubicin, and K562/G01 were cultured in the complete RPMI 1640 media containing 2 μM imatinib. KG-1a and KG-1 cells were cultured in IMDM supplemented with 20% FBS and 1% penicillin/streptomycin.

### MiRNA array

Total RNA was extracted using the miRCURY RNA Isolation Kit (EXIQON, Vedbaek, Denmark) according to the manufacturer's protocol. The samples were labeled using the miRCURY™ Hy3™/Hy5™ Power labeling kit and hybridized on the miRCURY™ LNA Array (v.18.0, EXIQON). Subsequently, the slides were washed and scanned using the Axon GenePix 4000B microarray scanner. Images were then imported into GenePix Pro 6.0 software (Axon Laboratory) for grid alignment and data extraction. Expressed data were normalized using the Median normalization, and significant differentially expressed miRNAs were identified through Volcano Plot filtering. Finally, hierarchical clustering was performed to show distinguishable miRNA expression profiling among samples. Differentially expressed miRNAs were further determined using TaqMan gene expression assays according to the manufacturer's protocol (Life Technologies, Carlsbad, USA).

### Ectopic expression of miRNA by lentiviral transduction

The lentiviral vectors carrying miR-99a or Ctrl were purchased from GeneCopoeia (Rockville, USA), and remolded with the SFFV promoter (Synbio Tech, Suzhou, China). Cell lines and LSCs isolated from BM of AML patients were transduced at a multiplicity of infection (MOI) of 30. EGFP positive cells were sorted by flow cytometry.

### Cell proliferation and cell cycle analyses

THP-1 or K562 cells transduced with miR-99a or Ctrl vectors were counted manually or stained with MTT solution (Roche, Basel, Switzerland) according to the manufacturer's protocol. Cells were also labeled with CFSE (Life Technologies) as described previously with slight modifications [43]. In brief, a total of 1 × 10^6^ cells were washed and stained in 1 mL of PBS containing 5 μM CFSE dye for 20 min at room temperature (RT) in darkness. Cells were quenched with 5 mL of culture media containing at least 1% protein for 5 min, washed twice, and then permeabilized with 2% paraformaldehyde (PFA). Cells were collected at different time points (0 hrs, 24 hrs, and 48 hrs) and analyzed by flow cytometry. Cell cycle was analyzed after propidium iodide (PI, Sigma-Aldrich) staining according to the manufacturer's instructions. Data were analysed using ModFit Software (Verity Software House, USA).

### Chemo-sensitivity assay

Cells were seeded into 96-well plates in the presence or absence of drugs for 72 hrs, and then incubated with MTT solution for 4 hrs and solubilized overnight in 10% sodium dodecyl sulfate (SDS) solution. Luminescence was measured using Multi-Mode Microplate Reader (BioTek, USA). The data were graphically displayed using GraphPad Prism version 5.0 (GraphPad Software, USA). IC_50_ was determined by a nonlinear regression model with a sigmoidal dose response in GraphPad. Doxorubicin and imatinib were purchased from Sigma-Aldrich, and Ara-C was from Pfizer (New York, USA).

### CFC and limiting-dilution assays

CFC assay was performed by plating primary LSCs transduced with viruses carrying miR-99a or Ctrl vectors in methylcellulose medium (MethoCult H4434, StemCell Technologies, Vancouver, Canada) at 4000 cells/mL in 24-well plates. Cell lines transduced with miR-99a or Ctrl vectors were pretreated with different drugs for 24 hrs, followed by plating cells into 24-well plates in 0.5 mL of methylcellulose medium (MethoCult H4230, StemCell Technologies) at 1000 cells/mL. After incubation at 37°C for 7-10 days, the number of colonies was counted. To evaluate the frequency of surviving cells after treatment, serially diluted cells (1000 down to 4 cells/well by 1:2.5 dilutions) were added into 96-well plates in 20 replicates. If no cell growth was observed after two weeks, this well was considered as negative. The frequency of resistant cells was estimated by linear regression analysis and Poisson statistics using Extreme Limiting Dilution Analysis software.

### Quantitative real-time PCR

Total RNA was obtained using TRIzol regent (Life Technologies) and reverse transcribed to cDNA using ImProm-II™ Reverse Transcription Kit (Promega, Madison, USA). The mRNA expression levels were measured on a 7900 real-time PCR instrument (Applied Biosystems). The primers used are listed in Table S1. The relative expression level of mRNA was calculated using the 2^−ΔΔт^ method adjusted by GAPDH as an internal control.

### Western blot analysis

Primary antibodies used for Western blots were CDKN2A/p14ARF (ab3642, abcam), p16 (ab51243, abcam), p-p53 (9286S, Cell Signaling Technology) and GAPDH (2118S, Cell Signaling Technology). Western blots were visualized using ECL detection reagents (Millipore).

### Animal study

Functional validation of enriched LSCs was done in *NOD/SCID* mice intrafemorally after irradiation with 300 cGy. The fraction of human CD45 and CD33 positive cells in BM was analyzed by flow cytometry. Subcutaneous AML models were established by injecting 1×10^7^ THP-1/miR-99a or THP-1/Ctrl cells into the right flank of five-week-old female nude mice. The tumor size was measured every other day throughout the study. The tumor volume was calculated by the following equation: tumor volume = (A × B^2^) × (1/2), where A was the longer dimension and B was the shorter dimension. When the tumor volume reached 200 mm^3^, mice were randomly divided into two groups. The mice in Ara-C group were treated with a daily intraperitoneal injection of 50 mg/kg Ara-C for a week, and control group was treated with vehicle control. Eight days post treatment, mice were euthanized and tumors were excised and embedded in paraffin for H&E staining. The tumor inhibition rate was calculated based on tumor volume in Ara-C group relative to the control group as follows: Tumor inhibition rate (%) = (1- tumor volume of treatment group/tumor volume of the control group) × 100%. Moreover, the tumor growth during the treatment was defined as the increase in tumor volume (mm^3^/day) in each mouse after drug injection. All animal experiments were approved by the institute's Animal Research Committee.

### Bioinformatic analyses

Total RNA of THP-1/Ctrl and THP-1/miR-99a was extracted from biological triplicate samples by TRIzol reagent and purified using RNeasy Mini Kit (Qiagen, Germany) including DNase digestion step. Microarray experiments were conducted by using GeneChip^®^ Human Gene 2.0 ST Arrays (Affymetrix GeneChip®, USA). Raw data (CEL files) was normalized using the robust multiaverage method. Genes differentially expressed were identified by the random variance model (RVM) t-test, with a threshold of *p* <0.05 and fold-change ≥1.2. Gene expression patterns were determined by cluster analysis based on the median using the Cluster and Java Treeview software.

Based on the GO Database and the Kyoto Encyclopedia of Genes and Genomes (KEGG) Database, GO and pathway analyses of differentially expressed genes were determined using DAVID bioinformatics resources. Significant differences were calculated with Fisher's exact test. A *p*-value of <0.05 was considered significant. GSEA was performed based on the Molecular Signatures Database (MSigDB) [44]. The LSC maintenance set used was published by Somervaille et al. [45], and MYC core module set was from Myc regulatory network [46].

Gene signal transduction networks were established to illustrate the inter-gene signaling between differentially expressed genes based on the IPA database (Qiagen), to identify key regulatory genes downstream of miR-99a. A list of potential targets of miR-99a was created by overlapping significant down-regulated genes in the microarray data with the target genes predicted by three databases: miRTarBase (http://mirtarbase.mbc.nctu.edu.tw), TargetScan (http://www.mirbase.org), and miRanda (http://www.ebi.ac.uk/enright-srv/microcosm). Downregulated predicted targets were then connected to the enriched genes in the significant networks by IPA, and targets corresponding to the higher number of genes or proteins were selected as candidate targets of miR-99a in this study.

### Statistical analyses

Statistical analyses were performed using GraphPad Prism 5.0 and SPSS 21.0 (IBM, Armonk, USA). A unpaired t-test or Mann-Whitney test was used for two group comparisons, and ANOVA analysis was used to determine differences among three or more groups. OS of AML was defined as the time from diagnosis of AML to death or was censored at the last follow-up. EFS was defined as the time from diagnosis of AML to the first adverse event such as induction failure, relapse, and death from any cause. Statistical analyses for OS and EFS were performed by Kaplan–Meier analysis and log-rank test. To determine the precision of the results, 95% confidence intervals (95% CIs) for the variables of interest were calculated. The number of biologically-independent replicates, and significance levels were shown in the figure legends. A *p*-value of < 0.05 was considered significant.

## SUPPLEMENTARY MATERIALS FIGURES AND TABLE


