# Supplementary material for: Upregulation of miR-99a is associated with poor prognosis of acute myeloid leukemia and promotes myeloid leukemia cell expansion
Source: Oncotarget. 2016 Oct 27;7(47):78095–109. doi: 10.18632/oncotarget.12947 (PMC5363646; doi:10.18632/oncotarget.12947)
Supplement: Supplementary file 1 [file oncotarget-07-78095-s001.pdf]

Upregulation of miR-99a is associated with poor prognosis of acute myeloid leukemia and promotes myeloid leukemia cell expansion

SUPPLEMENTAL FIGURES AND TABLE

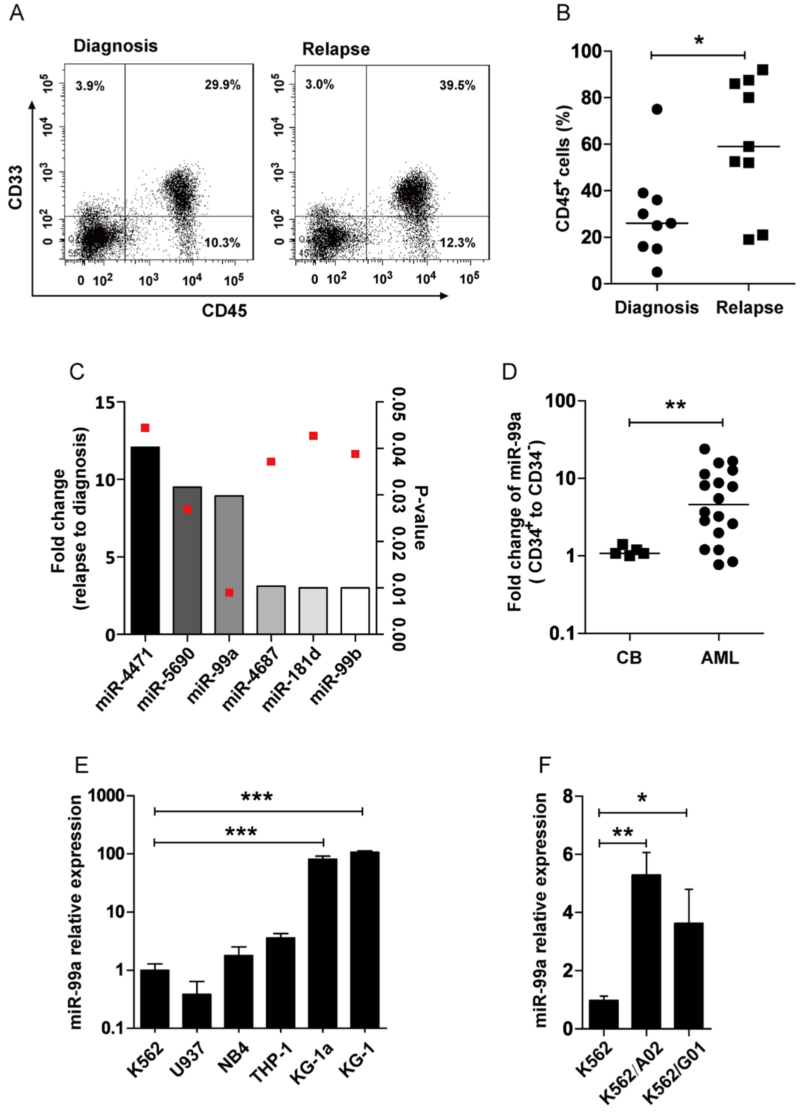

Supplementary Figure S1: A-B. Functional validation of LSCs enriched from diagnosis and relapse patient with AML in *NOD/SCID* mice (n= 9 mice/group). The fraction of human CD45 and CD33 positive cells in the BM of mice was analyzed by flow cytometer. Black lines indicated median. Data represented one of two independent experiments, *$p<0.05$. **C.** Fold change of upregulated miRNAs in LSCs obtained at relapse relative to that collected at the time of initial diagnosis. Red dots indicated p value. **D.** Fold change of miR-99a within LSCs relative to paired non-LSCs in AML patients, compared to that within CB-HSPCs relative to paired CB $CD34^-$ cells by qPCR analyses. The data were analyzed by Mann Whitney U test, and the black line indicated median. (n=18 for AML and n=5 for CB). U6 was used as the endogenous reference gene, **$p<0.01$. **E.** Relative expression levels of miR-99a within myeloid leukemia cell lines by qPCR analysis. Data are presented as mean ± SD and represented triplicate wells from one of two independent experiments. U6 was used as the endogenous reference gene. ***$p<0.001$, 1-way ANOVA. **F.** Relative expression levels of miR-99a within chemoresistant myeloid leukemia cells by qPCR analysis. Data are presented as mean ± SD and represented triplicate wells from one of two independent experiments. U6 was used as the endogenous reference gene. *$p<0.05$, **$p<0.01$, 1-way ANOVA.

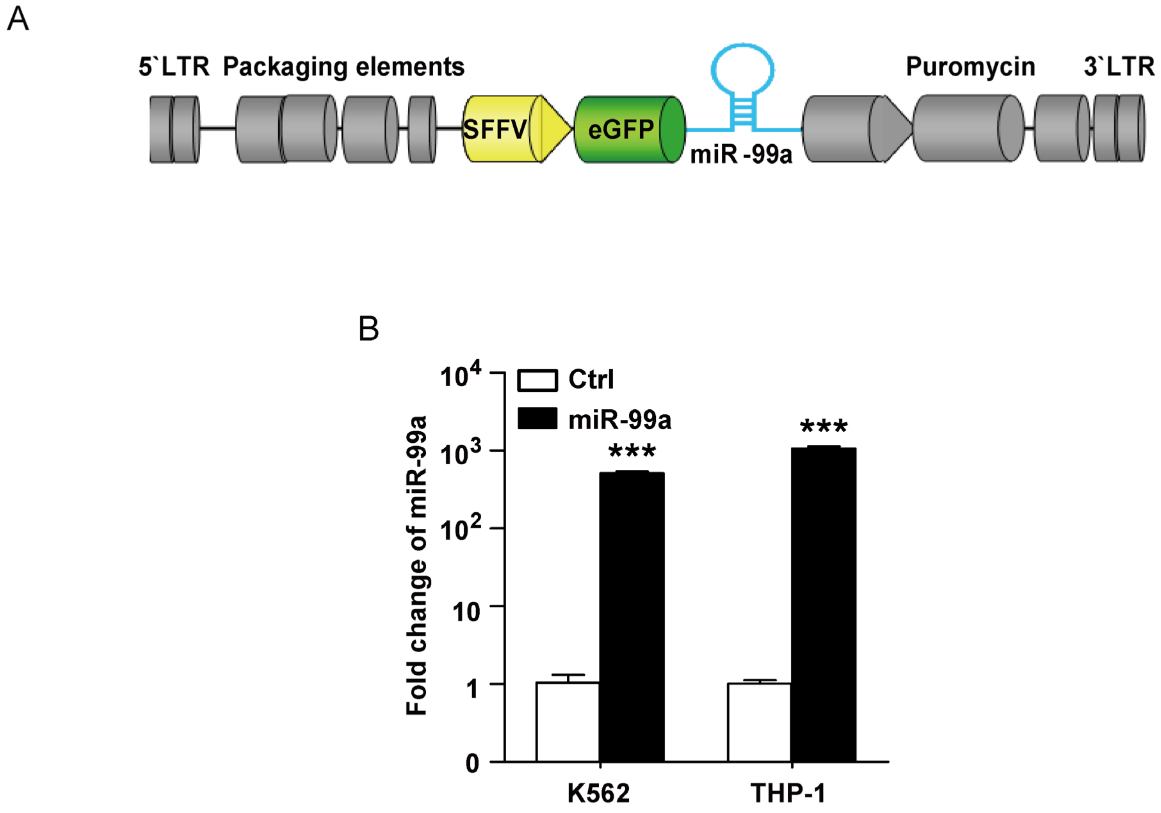

Supplementary Figure S2: A. Schematic representing the lentiviral construct for overexpression of miR-99a. The lentiviral vectors were tagged with enhanced green fluorescent protein (eGFP) and driven by an SFFV promoter. **B.** Relative expression levels of miR-99a in transduced cells by qPCR. Data are represented as the mean ± SD and represented triplicate wells from one of two independent experiments. U6 was used as the endogenous reference gene. ***p<0.001, 2-tailed Student's *t* test.

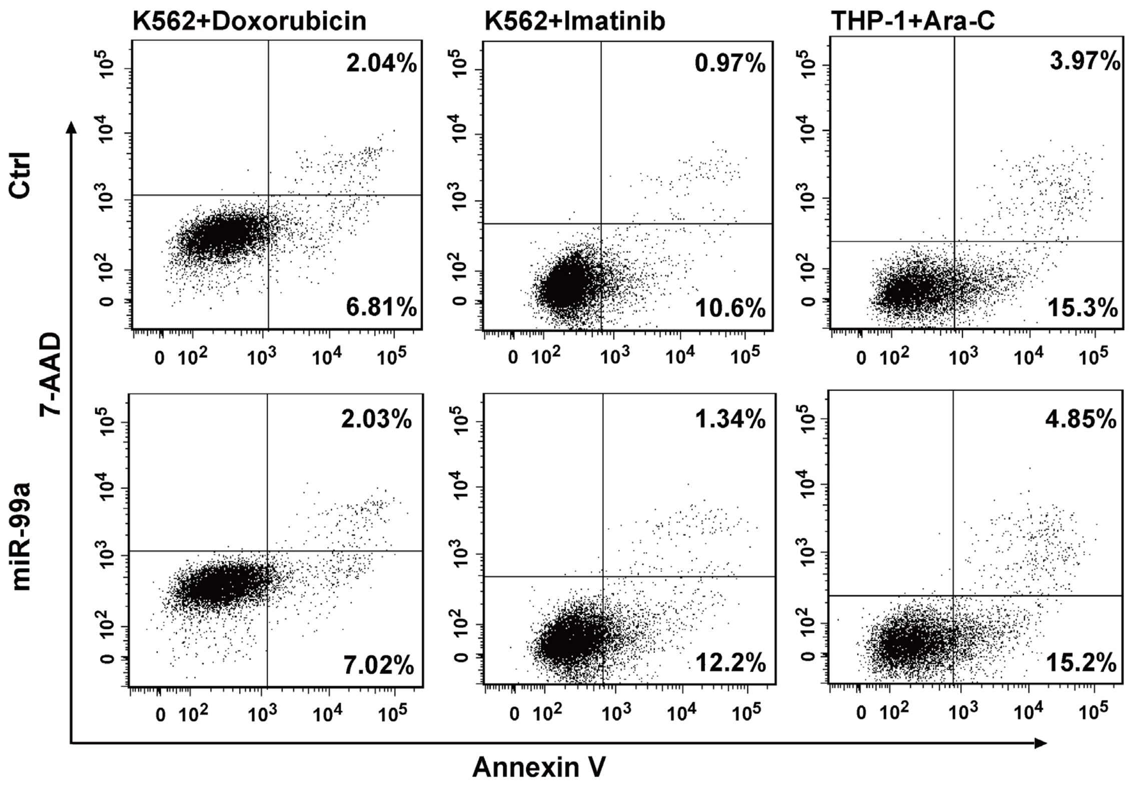

Supplementary Figure S3: Representative flow plots for apoptosis analysis with Annexin V and 7-AAD staining of Ctrl and miR-99a cells after drug treatment. Data represented one of two independent.

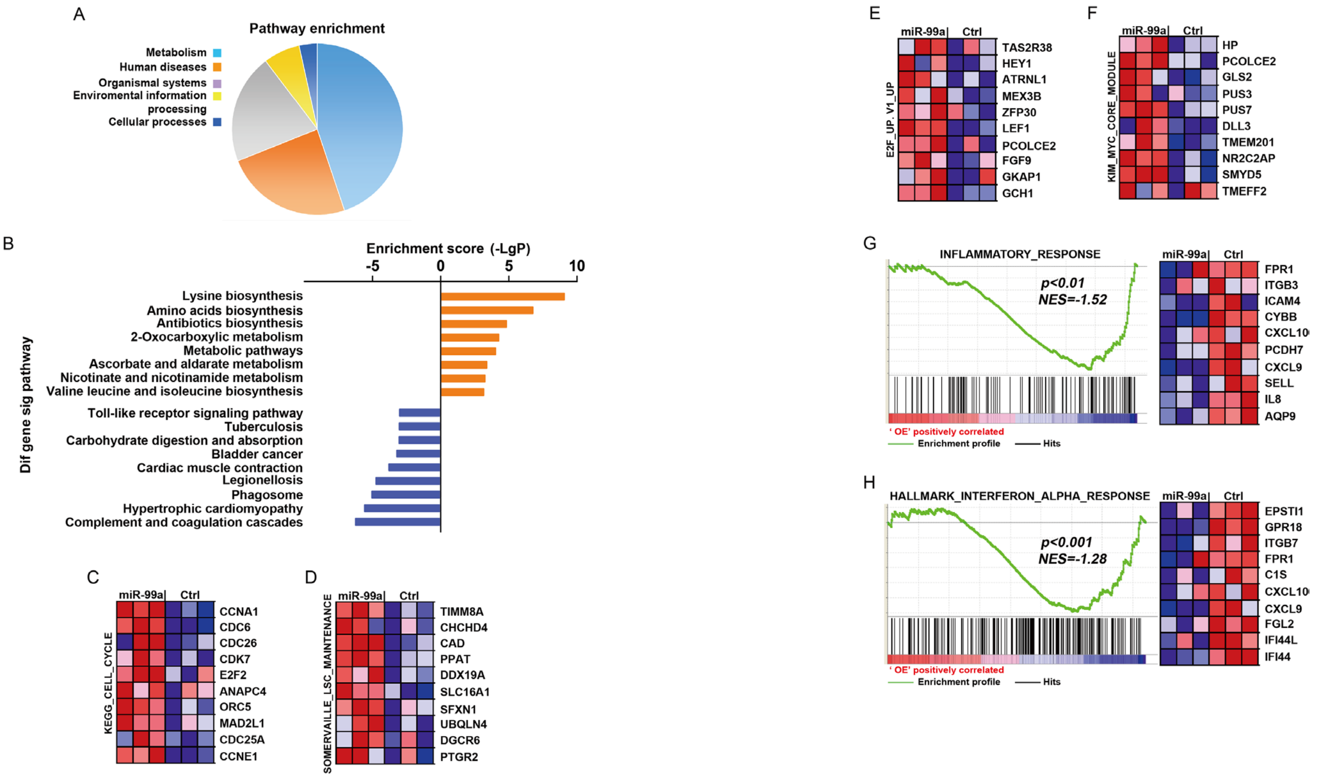

Supplementary Figure S4: A. Top pathway classification of microarray data. **B.** Pathway enrichment analysis of microarray data. Selected significant ontology terms are shown. **C-F.** Heat map of the GSEA plot showing the top 10 genes as described for Figure 5C-5F. KEGG cell cycle gene set (C), the LSC maintenance signature gene set (D), E2F-induced upregulated gene set (E), and the MYC core module gene set (F). **G-H.** GSEA plots are showing the enrichment of inflammatory response (G) and interferon alpha response (H). Heat maps show the top 10 genes. The normalized enrichment score (NES) and *p* values are shown.

Supplementary Table S1: List of primer sequences

RT-PCR

Primer	Sequence (5' to 3')
GAPDH-F	GAAGGTGAAGGTCGGAGTC
GAPDH-R	GAAGATGGTGATGGGATTTC
CTDSPL-F	CTGAAGCGGCCACATGTG
CTDSPL-R	CCAGCGGTCTAGGAGGTCA
PPP3CA-F	ATCCACACGAGGTTTTCCAT
PPP4CA-R	GGCAATTGATCCCAAGTTGT
PRDM1-F	ACGTGTGCCCTTTGGTATGT
PRDM1-R	GATGGCGGTACTTCGGTTC
PROX1-F	CAACATCTTTGCCTGCGATA
PROX1-R	ACAGGGCTCTGAACATGCAC
JUN-F	GTCCTTCTTCTCTTGCGTGG
JUN-R	GGAGACAAGTGGCAGAGTCC
CCNE1-F	TCTTTGTCAGGTGTGGGGA
CCNE1-R	GAAATGGCCAAAATCGACAG
CDKN2A-F	GGGTCGGGTGAGAGTGG
CDKN2A-R	GCCGCTTCCTAGAAGACCAG
E2F1-F	TCTCGGCCAGGTACTGATG
E2F1-R	ACCCTGACCTGCTGCTCTT
FOXM1-F	CTCTCAGTGCTGTTGATGGC
FOXM1-R	GGAGAATTGTCACCTGGAGC
SPIB-F	CCAGCAGGAACTGGTACAGG
SPIB-R	ACTTACCGTTGGACAGCCCT
